# Sterilizing the Unfit

**Published:** 1913-03

**Authors:** G. Henri Bogart

**Affiliations:** Paris, Illinois


For Texas Medical Journal.

### Sterilizing the Unfit.

BY DR. G. HENRI BOGART, PARIS, ILLINOIS.

Once more I desire to crave the indulgence of the readers of the "Red Back" while I talk a little along the thought of sterilizing the unfit.

At the last session of the Legislature of Texas, some of those who look to the future sought to write this law into the statutes of the great Lone Star State, that empire that is making so much progress towards development, and whose citizens are climbing towards a higher humanity.

The bill passed the House but died on the calendar in the Senate—for want of time. The last session was the one in which the best interests of legislation had to play the second fiddle to the game of politics; a Presidential campaign was on the boards. Then, too, we have rarely secured such a law without at least a second campaign. The first era has to be education.

Then, too, the wonderfully active growth of our American folks in knowing and far more in desiring to know the facts of growing humans with at least a tithe of the interest and intelligence bestowed upon "dumb-driven cattle" has prepared the general public for the receptiveness of the measure.

There are some splendid societies on social and racial lines in Texas, and I am assured that they are fully alive to this tremendously potent and beneficent measure for the future uplift of the race, and their influence will be exerted for its success this time. [Bill now before the Legislature.—ED.]

The influence of the TEXAS MEDICAL JOURNAL (the famous "Red Back") and its intensely earnest editorial management has exerted a wide educational influence, not only through the medical profession, but among thinking men and women as well.

I wish then to bring you this message: Texas will certainly take place as another star in the galaxy of Commonwealths in which crime and degeneracy and insanity shall be mercifully prevented. I shall urge every man and evey woman who reads this paper to add personal influence to induce his individual member of the Legislature to act intelligently. Whenever I see a debased degenerate, a suffering epileptic, or a crime-diseased sufferer in the toils of an inherent degradation, there is a thrill of joy actuates me that I have been instrumental in saving thousands from such cruel fate, in that they will never be born. Can not you, each and all of you, unite with me in this pleasure?

And now I would as well add a few words of caution. Pardon an illustration. A colony marooned on a small island would find little use for a shipload of gold unless they could use it, and the wanderer on the desert veldt would willingly barter a handful of diamonds for a draught of water. Nothing is of benefit only as it may be used.

A sterilization law that would not operate to curtail the birth of those foredoomed to evil is worse than no law. It is as though one went to war with a sword of lath.

Four States have laws on this subject which had better never have been written, in that their application is confined to those convicted of the third felony, and in New Jersey even then the subject must first be tried by a court and jury.

In Indiana a wording of the law leaves the ultimate decision as to the operation to a non-professional board of trustees, and consequently some of the institutions in which there is most need have no operations.

In Connecticut the operation is confined to one insane and two penal institutions, and, by a fault in phraseology, the female subjects are subjected to castration by ovariotomy instead of sterilization [by ligating and cutting the Fallopian tubes.—ED.].

Only one State has in so far passed a perfect institutional law, and that is Iowa. This law is mandatory in *all* institutions. It fully describes the classes subject to operation. It forces operation upon the confirmed habitual criminal and the sexual pervert. It leaves the operation in the hands of trained specialists, in conjunction with trained criminologists, in the Board of Pardons. It adds the proper control to unrestricted operation. This law will be found on page 387 of the TEXAS MEDICAL JOURNAL for April, 1912.

Things do not just happen; they are done.

As a final word I am begging that you, reader, constitute yourself into a *doer*.

Let us put Texas clear up head, and save our children and our neighbor's children the ever-increasing burden of crime, insanity and inherited evil, while we save the poor degenerates the living hell of existence.

